# A Proposed Classification of ICD-11 Severity Degrees of Personality Pathology Using the Self and Interpersonal Functioning Scale

**DOI:** 10.3389/fpsyt.2021.628057

**Published:** 2021-03-18

**Authors:** Dominick Gamache, Claudia Savard, Philippe Leclerc, Maude Payant, Nicolas Berthelot, Alexandre Côté, Jonathan Faucher, Mireille Lampron, Roxanne Lemieux, Kristel Mayrand, Marie-Chloé Nolin, Marc Tremblay

**Affiliations:** ^1^Department of Psychology, Université du Québec à Trois-Rivières, Trois-Rivières, QC, Canada; ^2^Department of Educational Fundamentals and Practices, Université Laval, Quebec City, QC, Canada; ^3^Department of Psychology, Université du Québec à Montréal, Montreal, QC, Canada; ^4^Department of Nursing Sciences, Université du Québec à Trois-Rivières, Trois-Rivières, QC, Canada; ^5^School of Psychology, Université Laval, Quebec City, QC, Canada; ^6^Centre intégré universitaire de santé et de services sociaux de la Capitale-Nationale, Quebec City, QC, Canada

**Keywords:** personality disorder, ICD-11 classification of personality disorders, dimensional models of personality disorders, degree of severity, self and interpersonal dysfunction

## Abstract

**Background:** The 11th version of the World Health Organization's International Classification of Diseases (ICD-11) has adopted a dimensional approach to personality disorder (PD) nosology. Notably, it includes an assessment of PD degree of severity, which can be classified according to five categories. To date, there is no gold standard measure for assessing degree of PD severity based on the ICD-11 model, and there are no empirically-based anchor points to delineate the proposed categories. With the operationalization of PD degrees of severity in the ICD-11 PD model now being closely aligned with Criterion A of the DSM-5 Alternative Model for Personality Disorders (AMPD), sharing a focus on self and interpersonal dysfunction, self-report instruments developed for the latter model might prove useful as screening tools to determine degrees of severity in the former.

**Methods:** The Self and Interpersonal Functioning Scale, a brief validated self-report questionnaire originally designed to assess level of personality pathology according to the AMPD framework, was used to derive anchor points to delineate the five severity degrees from the ICD-11 PD model. Data from five clinical and non-clinical samples (total *N* = 2,240) allowed identifying anchor points for classification, based on Receiver Operating Characteristic curve analysis, Latent Class Analysis, and data distribution statistics. Categories were validated using multiple indices pertaining to externalizing and internalizing symptoms relevant to PD.

**Results:** Analyses yielded the following anchor points for PD degrees of severity: No PD = 0–1.04; Personality Difficulty = 1.05–1.29; Mild PD = 1.30–1.89; Moderate PD = 1.90–2.49; and Severe PD = 2.50 and above. A clear gradient of severity across the five categories was observed in all samples. A high number of significant contrasts among PD categories were also observed on external variables, consistent with the ICD-11 PD degree of severity operationalization.

**Conclusions:** The present study provides potentially useful guidelines to determine severity of personality pathology based on the ICD-11 model. The use of a brief self-report questionnaire as a screening tool for assessing PD degrees of severity should be seen as a time-efficient support for clinical decision and treatment planning.

## Introduction

The field of personality disorders (PDs) is moving decisively toward a dimensional conceptualization of personality pathology. Shortcomings of the traditional, categorical method for PD classification have been well-documented [e.g., excessive comorbidity among disorders, heterogeneity within each category, inadequate coverage of PD presentations with an overreliance on “Not otherwise specified” diagnosis, lack of validity of diagnostic categories; e.g., (1, 2)]. It is widely believed that the adoption of a dimensional model of PDs will address these issues, and that a dimensional framework is more consistent with available empirical evidence on the nature of these disorders [e.g., (3–5)].

While calls for moving the field toward a dimensional paradigm are not new [e.g., (6–8)], it is only recently that changes have actually been implemented in the most recent versions of both the *Diagnostic and Statistical Manual for Mental Disorders* (DSM) and the *International Classification of Diseases* (ICD). An Alternative Model for Personality Disorders (AMPD) was introduced in Section III of the fifth edition of the DSM (9); it was meant to replace the traditional categorical PD model but was ultimately relegated to Section III by the APA Board of Trustees, awaiting further research. The AMPD includes two main components. Criterion A was proposed as an indicator of the level of personality pathology severity; it includes four elements that are believed to be closely intertwined and focus on impairments in one's sense of self (Identity and Self-direction) and in interpersonal relationships [Empathy and Intimacy; (10)]. Criterion B includes 25 maladaptive personality traits hierarchically organized into five broader domains [Negative Affectivity, Detachment, Antagonism, Disinhibition, and Psychoticism; (11)]. The model also retains six specific personality disorders that can be diagnosed based on “algorithms”; in these algorithms, the presence of two or more Criterion A elements and of Criterion B traits specific to each disorder is necessary for PD diagnosis, making the AMDP a hybrid categorical-dimensional model. Research on the AMPD has burgeoned over the past years and has yielded very promising results [see (12) for a summary].

For its part, the eleventh version of the World Health Organization's (WHO) *International Classification of Diseases* (ICD-11) has resolutely adopted a dimensional approach to PD nosology [see (4) for a detailed timeline of the different steps that led to the final proposal]. The purpose of the new model was “to provide a classification that was easily understood, could be readily used by practitioners of all disciplines, and that allowed all people with personality disturbance to be recognized” [(4), p. 497]. In the retained model, PD is defined as a marked disturbance in personality functioning, leading to considerable personal and social disruption in most cases. The central manifestations of PD are impairments in self-functioning (e.g., identity, self-worth, self-direction) and/or problems in interpersonal functioning (e.g., developing and maintaining close and mutually satisfying relationships, understanding others' perspectives, managing conflict). Both may manifest in maladaptive (e.g., inflexible, dysregulated) patterns of cognition, affective experience and expression, and behavior. PD can be classified according to a gradient of severity, ranging from (a) “No Personality Disorder,” (b) “Personality Difficulty,” (c) “Mild Personality Disorder,” (d) “Moderate Personality Disorder,” to (e) “Severe Personality Disorder” (13, 14). Table 1 summarizes the main differences among the different degrees of severity. The PD diagnosis may also be specified using one or more “Trait domain qualifiers” (Negative Affectivity, Detachment, Dissociality, Disinhibition, and Anankastia); an additional qualifier for Borderline Pattern may also be used. Of note, initial proposals for ICD-11 PDs did not focus on self and interpersonal dysfunction to define degree of severity, and were more closely aligned with the “British zeitgeist” of PD (15) in which personality pathology tends to be associated with potential of harm to self and others; they also did not include the Borderline specifier. It is only after some vocal opposition was expressed (16) that the initial proposal was amended.

**Table 1:** Main differences among the ICD-11 degrees of severity for personality disorders.

**Personality difficulty**	**Mild personality disorder**	**Moderate personality disorder**	**Severe personality disorder**
– Presence of personality characteristics that may affect treatment or health services but fall short of a proper PD diagnosis.	– Disturbances only affect some areas of functioning of the self, or affect all areas but are of mild severity.	– Disturbances affect multiple areas of functioning of the self and are of moderate severity.	– Severe disturbances in multiple areas of functioning of the self.
– Difficulties are expressed only intermittently or at a low level of intensity.	– Some problems are noted in relationships or in performance/social roles, but the individual is able to maintain some of them.	– Marked problems (e.g., conflict, avoidance, extreme dependency) are noted in most relationships, and performance in most social/occupational roles is affected to some degree.	– Serious problems affect virtually all relationships, and the individual is unable or unwilling to perform expected social and occupational roles.
– Difficulties are insufficiently severe to cause significant disruption in social, occupational, and interpersonal relationships or may be limited to specific relationships/situations.	– Not typically associated with significant harm to self or others.	– Sometimes associated with harm to self or others.	– Often associated with harm to self or others.
	– May be associated with substantial distress or with limited to circumscribed impairment in important areas of functioning.	– Associated with marked impairment in most important areas (although functioning in circumscribed areas may be preserved).	– Associated with severe impairment in all (or nearly all) important areas of life.

*Adapted from Bach and First (13) and World Health Organization (14). ICD-11, 11th revision of the International Classification of Diseases*.

Previous research has repeatedly shown that the global degree of PD severity, which will be the focus of this study, predicts a number of negative outcomes over and beyond PD categories. Indeed, general PD severity appears to be a strong predictor of current or future adjustment [e.g., (17–20)]. It also accounts for the comorbidity among categorical PD diagnoses (21) and appears to be sensitive to change (22). Moreover, it may provide valuable information for guiding intensity of clinical treatment [e.g., (13, 23)]. To this day, however, empirical work aiming to operationalize the ICD-11 degrees of severity has been scarce, in contrast with the AMPD Criterion A for which multiple self-report and clinician-rated measures have been developed to assess level of PD severity [see (24, 25) for a summary]. One notable exception is the Standardized Assessment of Severity of Personality Disorder [SASPD; (26)], which was specifically developed to assess the ICD-11 severity of PD. It includes nine items pertaining to traits from each of the five ICD-11 trait model domains; respondents are asked about the impact of a particular problem (e.g., acting on impulse, worrying) on their risk of harm to self and others and on their interpersonal functioning. Initial psychometric evaluation of the SASPD revealed good predictive ability for determining mild and moderate personality disorder severity, and high test-retest stability (26). However, the SASPD was developed based on the initial ICD-11 proposal for PD, and thus did not include elements pertaining to self and interpersonal deficits. In a comparative study of ICD-11 and DSM-5 Section III personality disorder models, McCabe and Widiger (27) found that the SASPD's convergence with the DSM-5 Section III model was improved when combined with the 12-item Level of Personality Functioning Scale-Brief Form [LPFS-BF; (28)], which assesses self and interpersonal impairments in line with the AMPD. McCabe and Widiger (27) concluded that the SASPD might benefit from a revision to include self and interpersonal deficits. In the same vein, in a study comparing the SASPD and the LPFS-BF in their relationships with external correlates, Bach and Anderson (15) outlined that the SASPD appeared to be more closely tied to the initial ICD-11 PD model, i.e., emphasizing risk of harm to self and others, while the LPFS-BF may have better sensitivity in detecting core personality disorder features (i.e., self and interpersonal pathology), which corresponds to the retained model. Furthermore, in a study of the psychometric properties of the SASPD in German non-clinical and clinical samples, Rek et al. (29) found mixed results for convergent and discriminant validity, calling into question its future usage as a screening tool of ICD-11-based degrees of PD severity.

These results, which highlight the potential shortcomings of the SASPD to capture essential features of the final ICD-11 PD model, along with positive results obtained using a self-report initially aimed to operationalize the AMPD, suggest that measures of the latter model might be useful in assessing ICD-11 PD degrees of severity. This “cross walk” strategy was also advocated by Bach and First [(13), p. 6], who stressed that “diagnostic information obtained from assessment tools developed for the DSM-5 AMPD model can be used for making an ICD-11 dimensional Personality Disorder diagnosis.” As aforementioned, there have been numerous self-report questionnaires developed to assess the AMPD that could be useful for that purpose (25). One of these instruments is the Self and Interpersonal Functioning Scale [SIFS; (30)], a 24-item measure originally developed based on the AMPD Criterion A conceptualization. It provides a global personality dysfunction score and four subscale scores, corresponding to AMPD elements (Identity, Self-direction, Empathy, and Intimacy). In its original validation study, meaningful patterns of associations with related psychological constructs (e.g., self-esteem, satisfaction with life, empathy, aggression, pathological narcissism, borderline symptomatology, and AMPD Criterion B domains) were reported. Confirmatory Factor Analysis yielded a second-order model, with four elements organized into a higher-order personality dysfunction factor, consistent with AMPD formulation. In an independent study, content validity analysis of the SIFS items also showed promising results, and the severity level assessed by its items makes it well-suited to study populations with greater psychopathology (25).

The purpose of the present study is to determine, based on the SIFS' global score (i.e., a general indicator of personality impairment), cutoff points corresponding to the five categories in the ICD-11 PD model. Although there might be some irony in “imposing” categories in a fundamentally dimensional framework (31), we believe that these categories are likely to provide clinicians and researchers with useful guidelines, e.g., for treatment planning and level of care assessment [e.g., (13, 23)]. Furthermore, we concur with Rek et al. (29) that accessible and time-efficient PD screening tools are crucial in a context in which these pathologies are too often overlooked during assessment. Providing guidelines for a screening of PD severity based on a short and validated self-report measure such as the SIFS might be a valuable contribution in this regard.

## Materials and Methods

### Participants and Procedure

A total of 2,240 adults, mainly French-speaking Canadians, were recruited in the Province of Quebec, Canada (84.6% women, *M*_age_ = 31.43, *SD* = 8.66, range 18–79). They were recruited in five distinct samples. The first three correspond to clinical samples. Sample 1 (*n* = 287) includes prospective PD patients with a more severe clinical presentation, recruited during the intake procedure at a specialized psychiatric outpatient clinic in the Quebec City area. Sample 2 (*n* = 249) includes prospective PD patients with a less severe clinical presentation, who were also recruited during the intake procedure from different outpatient treatment establishments in the Quebec City area. Both settings are public, and have a mandate of treating PD patients; in line with a stepped care approach [e.g., see (32)], those with more severe clinical presentations are referred to the first clinic for more intensive treatment (Sample 1), while those with less severe presentations are referred to other establishments who offer PD treatment programs but with a less intensive level of care (Sample 2). Sample 3 (*n* = 242) includes patients from two general private practice clinics located in Quebec City; these clinics use a common set of intake measures as part of a collaborative study. The last two samples correspond to non-clinical participants. Sample 4 (*n* = 1,200) includes female participants from a study of pregnant women's mental health. They were recruited through advertisement on social media (Facebook and Instagram). Finally, Sample 5 (*n* = 263) includes participants from the community recruited as part of the initial validation study of the SIFS (30). They were recruited through social media, online message boards, and institutional e-mail from two universities in the Province of Quebec. Supplementary Table 1 provides more detail on socio-demographic characteristics of these five samples.

### Measures

#### Identification of Cutoffs for the ICD-11 PD Severity Degrees

The Self and Interpersonal Functioning Scale (30), described above, was used in our main analyses to determine clinical cutoff points corresponding to the proposed ICD-11 anchors for PD severity, based on its global score (Cronbach's alpha [α] for the combined sample =0.91). Items are rated on a five-point scale (range 0–4). Descriptive statistics for all five samples are displayed in Supplementary Table 2.

#### External Validation of Degrees of Severity

Samples had different sets of self-report questionnaires, which were used in further analyses to validate the different anchor points established using the SIFS. Supplementary Table 2 displays the different questionnaires from each sample, along with their descriptive statistics (i.e., mean scores and internal consistency indices).

##### Samples 1, 2, 3, and 5

The Personality Inventory for DSM-5 was used in its 100-item version, the PID-5 Faceted Brief Form [PID-5-FBF; (33); French validation by Roskam et al. (34)] for Samples 1, 3, and 5, and in its 25-item version, the PID-5 Brief Form [PID-5-BF]; (35); French validation by Combaluzier et al. (36)] for Sample 2. It covers five domains of pathological personality functioning: Negative Affectivity, Detachment, Antagonism, Disinhibition, and Psychoticism. Items are rated on a four-point scale (range 0–3).

##### Samples 1 and 2

The 23-item version of the Borderline Symptom List [BSL-23; (37); French validation by Nicastro et al. (38)] assesses borderline PD symptomatology according to DSM Section II BPD diagnostic criteria, in addition to other affective experiences typical of borderline pathology (e.g., proneness to shame, self-criticism, mistrustfulness). Items are scored on a five-point scale (range 0–4).

The 28-item Brief Version of the Pathological Narcissism Inventory [B-PNI; (39); French validation by Diguer et al. (40)] was used to measure two dimensions of pathological narcissism: Grandiosity (e.g., inflated self-image, exploitative behaviors, fantasies of power and perfection) and Vulnerability (e.g., depleted self-image, shame/anger, interpersonal hypersensitivity). Items are scored on a six-point scale (range 0–5).

##### Sample 1 Only

The 12-item short-form Buss-Perry Aggression Questionnaire [BPAQ-SF; (41, 42); French validation by Genoud and Zimmerman (43)] covers four manifestations of aggression: Verbal, Physical, Anger, and Hostility. It also yields a global Trait Aggression score. Items are scored on a six-point scale (range 1–6).

The 28-item Interpersonal Reactivity Index [IRI; (44); French validation by Gilet et al. (45)] measures empathy and its components. Two of its subscales were used in the present study: Perspective Taking (the ability to adopt others' point of view), which assesses the cognitive component of empathy, and Empathic Concern (the motivation to care about others), which focuses on the affective component. Items are scored on a seven-point scale (range 1–7).

The 30-item Barratt Impulsiveness Scale [BIS-11; (46); French validation by Baylé et al. (47)] is designed to assess three components of impulsiveness: Attentional, Motor, and Non-planning. Items are scored on a four-point scale (range 1–4).

##### Sample 3 Only

The 14-item version of the Psychiatric Symptom Index [PSI; (48); French validation by Préville et al. (49)] covers core psychological symptoms (e.g., depression, anxiety, anger). Items are scored on a four-point scale (range 0–3).

The shortened 12-item Experiences in Close Relationship Questionnaire [ECR-12; (50)] assesses both dimensions of romantic attachment: Anxiety about relationship issues, and Avoidance (discomfort with closeness and interdependence). Items are scored on a seven-point scale (range 1–7).

##### Sample 4 Only

The 10-item Kessler Psychological Distress Scale [K-10; (51) French validation by Gravel et al. (52)] assesses anxious and depressive symptomatology. Items are rated on a five-point scale (range 1–5).

The 10-item Edinburgh Perinatal/Postnatal Depression Scale [EPDS; (53) French validation by Adouard et al. (54)] indicates the presence of depressive symptoms during pregnancy and in the year following childbirth. Items are scored on a four-point scale (range 1–4).

The 20-item Positive and Negative Affect Schedule [PANAS; (55); French validation by Gaudreau et al. (56)] covers the experience of feelings such as energy, enthusiasm, and inspiration (Positive Affect), as well as experiences such as fear, hostility, and shame (Negative Affect). Items are scored on a five-point scale (range 1–5).

Two subscales of the Dissociative Experiences Scale [DES; (57); French validation by Larøi et al. (58)], Absorption/Imaginative involvement (nine items) and Depersonalization/Derealization (six items), were used. Items are rated on an 11-point scale (range 0–10).

The 20-item Posttraumatic Stress Disorder (PTSD) Checklist for DSM-5 [PCL-5; (59); French validation by Ashbaugh et al. (60)] covers trauma-related symptoms aligned with the PTSD diagnostic criteria of the DSM-5. Items are rated on a five-point scale (range 0–4).

##### Sample 5 Only

The 19-item brief version of the Inventory of Personality Organization (IPO) validated by Verreault et al. (61) includes three scales from the original IPO (62): Identity Diffusion, Primitive Defenses, and Impaired Reality Testing, along with a Global Personality Organization score. Items are scored on a five-point scale (range 1–5).

The five-item Satisfaction with Life Scale [SWLS; (63); French validation by Blais et al. (64)] uses straightforward probes about participants' life satisfaction. Items are scored on a seven-point scale (range 1–7).

The 10-item Rosenberg Self-Esteem Scale [RSES; (65); French validation by Vallières and Vallerand (66)] is a unidimensional measure of global self-esteem. Items are scored on a four-point scale (range 1–4).

### Analytic Strategy

In a preliminary step, *t*-tests for independent samples were computed on the global SIFS score for men and women for each sample (with the exception of Sample 4 which only includes women), to rule out the need for separate cutoffs based on gender. The ensuing statistical analyses followed a four-step procedure. (a) The first step aimed at delineating participants with vs. without a personality disorder. This should establish a first threshold between the “No PD” and the “Personality Difficulty” groups, on the one hand, and the Mild-Moderate-Severe PD groups, on the other hand. In order to do so, we ran a Receiver Operating Characteristic (ROC) curve analysis, combining Samples 1 and 2 (PD patients) to form a first dichotomous groups (with PD), and combining Samples 4 and 5 (pregnant women and participants from the community) to form a second dichotomous group (without PD)^1^. Sample 3, recruited in private practice clinics, was excluded at this step, as the expected prevalence of PD in these clinical settings is uncertain. The ROC analysis allowed choosing a cutoff point to establish the presence of a PD; this cutoff was selected based on empirical considerations (i.e., optimal sensitivity-specificity based on Youden's index, Diagnostic odds ratio). It was also based on “clinical plausibility,” as the retained cutoff should yield a PD prevalence in non-clinical groups in the range observed in past epidemiological studies conducted in community samples. While the range of these estimates is quite large, from 4.4 (68) to 33.1% (69), the selected cutoff should ideally yield prevalence indices close to the median estimate of 11.5%, based on 14 major studies, reported by Morgan and Zimmerman (67).

(b) In a second step, delineation among the three categories where PD is present (Mild, Moderate, Severe) according to the ICD-11 model was established. This was computed using Latent Class Analysis (LCA) on the subsample of participants from all five samples identified as having a PD based on the threshold established in step (a). The SIFS total score was used as the sole latent indicator, and a predetermined number of three profiles was specified for the analysis. These profiles are thus expected to be established based on a gradient of severity; the Mild severity profile is expected to have more participants, while the Severe profile should have less, and the Moderate severity should be in between these two. Hallquist and Pilkonis (70), drawing on a previous work from Markon and Krueger (71), have commented on the potential use of LCA to determine severity degrees, suggesting that “when LCA supports the existence of two or more latent classes that differ by severity, the mean severity level for each class provides potential information about cut-points along a continuous severity dimension” (p. 229). Thus, means and standard deviations from the three profiles yielded by LCA were used to establish anchor points for the Mild, Moderate, and Severe categories.

(c) The third step involved delineating the “No PD” from the “Personality Difficulty” group. This latter group is defined in the ICD-11 PD model by the presence of long-standing difficulties in a person's way of experiencing and thinking about oneself, others, and the world; in contrast to PD proper, however, individuals with Personality Difficulty only show intermittent, or low intensity, manifestations of these difficulties in the cognitive, emotional, or behavioral domains (14). Thus, individuals with Personality Difficulty might be expected to be found in prospective patients referred to PD clinical programs in Samples 1 and 2, but who did not reach the diagnostic threshold for a PD. The delineation between No PD and Personality Difficulty was based on mean and standard deviation (i.e., within ≤ 1.0 *SD*) for these individuals.

(d) Finally, once anchor points were established for all five PD and non-PD categories, the last step involved their validation based on a set of external variables relevant for PD and general psychosocial functioning. The five samples included different sets of mostly non-overlapping self-report measures, tailored to their clinical context (e.g., more measures of externalizing pathology and potential for harm in the most severe samples). These measures should provide a wide range of clinical and functioning variables for validation. Samples were tested separately, using Kruskall-Wallis analysis (two-tailed, with Bonferroni's correction for multiple comparisons) to contrast categories, as the number of participants per category was likely to show marked differences. In Samples 1 and 2, the “No PD” category was omitted from analyses as it was expected to have a very low prevalence in these groups^2^. The same goes for the “Severe PD” group in Samples 3, 4, and 5. Valid PD severity categories would be expected to show a gradient of severity (i.e., minimal pathology in the No PD group, and maximal pathology in the Severe group, with increments between intermediate categories). Contrasts between contiguous/consecutive categories (e.g., between No PD and Personality Difficulty, or between Moderate and Severe PD) were considered a stringent test of the discriminative power of the cutoffs established through steps (a) to (c).

## Results

In a preliminary step, gender differences for the SIFS total score were ruled out for each sample: Sample 1 = *M*_women_ = 1.89, *SD* = 0.62; *M*_men_ = 1.92, *SD* = 0.58; *t*_(285)_ = −0.48, *p* = 0.63; Sample 2 = *M*_women_ = 1.66, *SD* = 0.61; *M*_men_ = 1.75, *SD* = 0.56; *t*_(246)_ = −1.18, *p* = 0.24; Sample 3 = *M*_women_ = 1.09, *SD* = 0.52; *M*_men_ = 1.19, *SD* = 0.60; *t*_(240)_ = −1.29, *p* = 0.20; Sample 5 = *M*_women_ = 0.97, *SD* = 0.48; *M*_men_ = 1.01, *SD* = 0.50; *t*_(261)_ = −0.57, *p* = 0.57. These results allowed determining common cutoffs for men and women in the ensuing analytic steps.

### Delineation Between Participants With vs. Without PD

**Figure 1:**
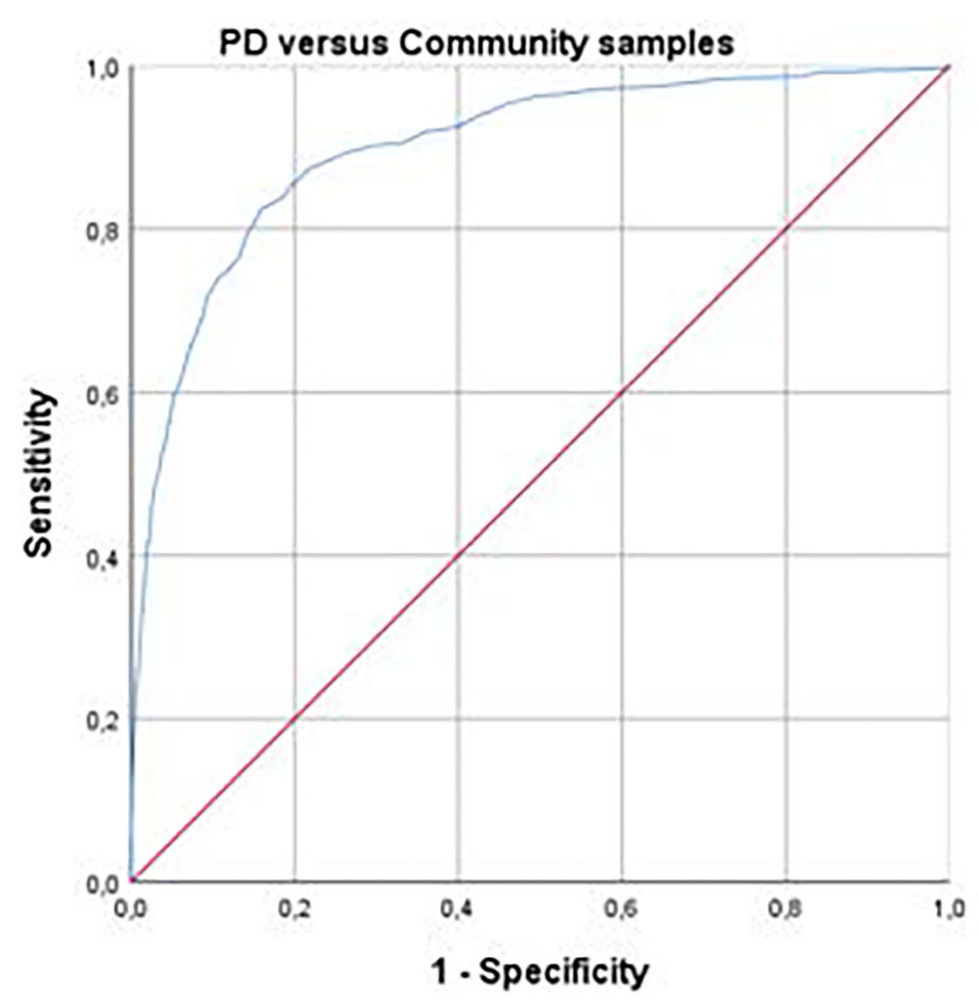
Receiver Operating Characteristic (ROC) curve using Self and Interpersonal Functioning Scale total score to discriminate between personality disorder and community samples. Personality disorder samples = combination of samples 1 and 2 (*n* = 536); Community samples = combination of samples 4 and 5 (*n* = 1,463). Area under curve = 0.90 (Confidence internal [0.88–0.92], Standard error = 0.01).

ROC analysis was performed using the SIFS total score as predictor of belonging to a PD group (Samples 1 and 2) vs. a non-clinical group (Samples 4 and 5; see Figure 1). Area under the curve was 0.90 (CI [0.88–0.92], *SE* = 0.01), an excellent accuracy with a large effect size (*d* = 1.84) according to established guidelines (72). Based on aforementioned criteria for cutoff selection, we chose 1.31 (rounded to 1.30; Sensitivity = 0.79, Specificity = 0.86, Diagnostic odds ratio = 23.23) as the cutoff between PD and absence of PD. This anchor point yielded the following prevalence for PD in our five samples: Sample 1 = 82.2%; Sample 2 = 71.3%; Sample 3 = 32.6%; Sample 4 = 11.2%; Sample 5 = 22.4%.

### Delineation Among the Mild, Moderate, and Severe PD Groups Based on Degree of Severity

LCA was run using Mplus version 8.4 (73) on participants (*n* = 713) with a PD according to the 1.30 cutoff established in step (a), with the standardized SIFS total score as the profiling variable, forcing the analysis to extract three classes from data. The entropy figure (0.81) suggested an adequate classification according to usual guidelines (a score between 0.8 and 1.0 is generally considered adequate). The three classes, Mild, Moderate, and Severe PD, included, respectively, 439 (61.6%; *M* = 1.59, *SD* = 0.17), 213 (29.9%; *M* = 2.18, *SD* = 0.18), and 61 (8.6%; *M* = 2.84, *SD* = 0.23) participants.

Using SIFS means and standard deviations of roughly +/– 1.5 units for the three classes generated by LCA, the following anchor points were established for the three degrees of PD severity: Mild = 1.30–1.89; Moderate = 1.90–2.49; Severe = 2.50 and above (see Table 2).

**Table 2:** Distribution of participants from the five samples according to ICD-11 degrees of severity thresholds established with the Self and Interpersonal Functioning Scale total score.

		**Clinical samples**			**Non-clinical samples**	
**Category**	**SIFS score range**	**Sample 1**	**Sample 2**	**Sample 3**	**Sample 4**	**Sample 5**
		**(*n* = 287)**	**(*n* = 249)**	**(*n* = 242)**	**(*n* = 1,200)**	**(*n* = 263)**
No PD	0–1.04	24 (8.4%)	32 (12.9%)	124 (51.2%)	927 (77.3%)	150 (57.0%)
Personality difficulty	1.05–1.29	21 (7.3%)	35 (14.1%)	33 (13.6%)	128 (10.7%)	53 (20.2%)
Mild PD	1.30–1.89	100 (34.8%)	95 (38.2%)	64 (26.4%)	126 (10.5%)	45 (17.1%)
Moderate PD	1.90–2.49	98 (34.1%)	62 (24.9%)	15 (6.2%)	18 (1.5%)	13 (4.9%)
Severe PD	2.50 and above	44 (15.3%)	25 (10.0%)	6 (2.5%)	1 (0.1%)	2 (0.8%)

*ICD-11, 11th revision of the International Classification of Diseases; SIFS, Self and Interpersonal Functioning Scale; PD, Personality disorder; Sample 1, Specialized psychiatric outpatient clinic for more severe PD; Sample 2, Outpatient treatment establishments for less severe PD; Sample 3, Private practice clinics; Sample 4, Pregnant women; Sample 5, Community participants*.

### Delineating Absence of PD From Personality Difficulty

Using the subsample of patients from Samples 1 and 2 who did not reach the threshold for PD (*n* = 123), we computed their mean SIFS score (*M* = 1.29, *SD* = 0.26) to determine the cutoff for Absence of PD vs. Personality Difficulty. Using an estimate of 1.0 *SD*, the final threshold between these two categories was established at 1.03 (rounded to 1.05); therefore, the Personality Difficulty anchor points correspond to 1.05–1.29 (see Table 2).

In sum, the final thresholds, corresponding to ICD-11 PD degree of severity categories and based on SIFS total score, are as follows: Absence of PD = 0 to 1.04; Personality Difficulty = 1.05 to 1.29; Mild PD = 1.30 to 1.89; Moderate PD = 1.90 to 2.49; and Severe PD = 2.50 and above. Table 2 shows how participants from the five samples included in the present study are distributed along the five categories.

### Validation of Categories With External Variables

Tables 3–7 display, for each sample, how participants from the five ICD-11 degree of severity categories differ across a number of external validation indices. Intergroup differences for the SIFS total score and elements are also presented but will not be considered as “external” comparators in the following analyzes as the ICD-11 categories are not statistically independent from these indices. A general pattern of results emerged in all five samples, as a clear gradient of severity across the five ICD-11 categories was observed. A high number of significant contrasts among PD categories were also observed.

**Table 3:** Comparisons among the ICD-11 personality disorders degrees of severity on external variables for sample 1 (specialized clinic for more severe personality disorders).

**Scale**	**Statistic**	**Personality difficulty**	**Mild PD**	**Moderate PD**	**Severe PD**	***H***
		**(*n* = 21)**	**(*n* = 100)**	**(*n* = 98)**	**(*n* = 44)**	
SIFS total	*M(SD)*	1.27 (0.13)	1.70 (0.20)	2.19 (0.20)	2.78 (0.23)	217.46^**^
	MR^b^	14.12_a_	74.60_b_	167.32_c_	240.05_d_	
SIFS identity	*M(SD)*	1.95 (0.49)	2.40 (0.56)	2.83 (0.50)	3.19 (0.46)	83.53^**^
	MR^b^	49.86_a_	100.33_b_	152.17_c_	198.26_d_	
SIFS self-direction	*M(SD)*	1.13 (0.44)	1.74 (0.63)	2.24 (0.60)	3.01 (0.66)	108.58^**^
	MR^b^	40.57_a_	97.84_b_	149.16_c_	215.06_d_	
SIFS empathy	*M(SD)*	0.74 (0.41)	1.02 (0.49)	1.61 (0.61)	2.36 (0.59)	120.57^**^
	MR^b^	57.02_a_	87.86_a_	154.02_b_	219.06_c_	
SIFS intimacy	*M(SD)*	0.90 (0.37)	1.39 (0.56)	2.10 (0.65)	2.80 (0.67)	127.77^**^
	MR^b^	40.10_a_	88.24_b_	159.86_c_	213.41_d_	
PID-5 negative affectivity	*M(SD)*	1.51 (0.48)	1.82 (0.59)	2.11 (0.51)	2.07 (0.55)	28.64^**^
	MR^b^	71.57_a_	115.36_ac_	153.71_b_	150.32_bc_	
PID-5 detachment	*M(SD)*	1.12 (0.63)	1.35 (0.60)	1.57 (0.50)	1.92 (0.56)	37.04^**^
	MR^b^	84.31_a_	111.36_a_	140.12_b_	183.58_c_	
PID-5 antagonism	*M(SD)*	0.38 (0.34)	0.47 (0.46)	0.77 (0.59)	1.23 (0.74)	44.10^**^
	MR^b^	93.48_a_	104.36_a_	143.77_b_	187.00_c_	
PID-5 disinhibition	*M(SD)*	0.98 (0.42)	1.36 (0.52)	1.65 (0.53)	2.05 (0.47)	65.67^**^
	MR^b^	56.60_a_	107.00_b_	144.88_c_	196.13_d_	
PID-5 psychoticism	*M(SD)*	0.46 (0.33)	0.79 (0.62)	0.98 (0.55)	1.22 (0.58)	35.56^**^
	MR^b^	70.81_a_	113.43_a_	145.67_b_	172.97_b_	
BSL-23	*M(SD)*	1.42 (0.59)	1.83 (0.90)	2.28 (0.83)	2.56 (0.79)	36.76^**^
	MR^b^	70.76_a_	111.29_a_	147.13_b_	171.15_b_	
PNI grandiose	*M(SD)*	1.71 (0.80)	2.01 (0.87)	2.52 (0.91)	2.77 (1.26)	30.59^**^
	MR^b^	85.21_a_	109.06_a_	148.76_b_	165.77_b_	
PNI vulnerable	*M(SD)*	1.66 (0.80)	2.05 (0.80)	2.69 (0.81)	3.24 (0.88)	69.24^**^
	MR^b^	66.19_a_	99.27_a_	150.05_b_	193.88_c_	
BPAQ total (trait aggression)	*M(SD)*	2.71 (0.92)	2.88 (0.90)	3.51 (1.02)	4.35 (0.96)	63.35^**^
	MR^b^	87.69_a_	98.53_a_	143.89_b_	199.01_c_	
BPAQ verbal aggression	*M(SD)*	2.48 (0.79)	2.54 (1.06)	3.14 (1.18)	3.75 (1.27)	32.85^**^
	MR^b^	102.52_ab_	105.62_b_	143.67_ac_	176.45_c_	
BPAQ physical aggression	*M(SD)*	2.29 (1.61)	2.17 (1.31)	2.68 (1.68)	3.69 (1.74)	25.33^**^
	MR^b^	110.69_a_	112.87_a_	133.53_a_	178.82_b_	
BPAQ hostility	*M(SD)*	2.95 (1.31)	3.53 (1.21)	4.17 (1.22)	5.01 (1.06)	54.94^**^
	MR^b^	74.31_a_	105.35_a_	142.76_b_	192.68_c_	
BPAQ anger	*M(SD)*	3.13 (1.34)	3.30 (1.39)	4.06 (1.25)	4.96 (1.16)	51.65^**^
	MR^b^	93.93_ab_	101.98_a_	141.52_b_	193.53_c_	
IRI perspective taking^a^	*M(SD)*	4.94 (0.88)	4.86 (1.12)	4.27 (1.06)	3.12 (1.25)	60.25^**^
	MR^b^	169.07_ab_	162.75_a_	124.36_b_	61.45_c_	
IRI empathic concern^a^	*M(SD)*	5.41 (0.88)	5.42 (0.97)	5.31 (1.29)	4.57 (1.28)	14.45^*^
	MR^b^	140.45_ab_	140.07_b_	139.86_b_	92.26_ac_	
BIS-11 attentional	*M(SD)*	2.20 (0.42)	2.40 (0.51)	2.66 (0.43)	3.00 (0.40)	57.24^**^
	MR^b^	75.40_a_	104.33_a_	142.62_b_	194.64_c_	
BIS-11 motor	*M(SD)*	1.85 (0.32)	2.17 (0.44)	2.41 (0.50)	2.89 (0.45)	72.27^**^
	MR^b^	57.50_a_	106.17_b_	140.71_c_	203.31_d_	
BIS-11 non-planning	*M(SD)*	2.29 (0.31)	2.47 (0.35)	2.63 (0.41)	2.92 (0.41)	47.21^**^
	MR^b^	75.90_a_	108.31_a_	141.36_b_	188.38_c_	

*ICD-11, 11th revision of the International Classification of Diseases; MR, Mean rank; SIFS, Self and Interpersonal Functioning Scale; PID-5, Personality Inventory for DSM−5 Faceted Brief Form (100 items); BSL-23, 23-item Borderline Symptoms List; PNI, Brief Version of the Pathological Narcissism Inventory; BPAQ, 12-item version of the Buss-Perry Aggression Questionnaire; IRI, Interpersonal Reactivity Index; BIS-11, Barratt Impulsiveness Scale (version 11)*.

a *Higher scores denote better functioning. For all other variables, higher scores denote more severe pathology*.

b *Mean rank with different subscripts (_a_,_b_,_c_,_d_) indicate significant post-hoc comparisons following a significant Kruskall-Wallis test, two-tailed, p < 0.05, using Bonferroni's correction for multiple comparisons*.

* *p < 0.01*;

** *p < 0.001*.

In the most severe sample (Sample 1), there was a remarkably neat break between the Mild and Moderate categories, as significant differences were observed for 16 out of 18 comparisons. There was also a clear delineation between the Moderate and Severe categories, with 13 out of 18 contrasts yielding significant results. Fewer differences (two) were observed between the Personality Difficulty vs. Mild categories. Of note, two comparators (PID-5 Disinhibition and BIS-11 Motor impulsivity) showed a high discriminant capacity, with significant differences across all four ICD-11 categories tested in that sample (see Table 3). The other PD sample (Sample 2) showed a similar pattern of results, with a clear gradient of severity across PD severity categories for all external variables, although less significant contrasts between contiguous categories were observed: four out of eight for Personality Difficulty vs. Mild PD (PID-5 Disinhibition and Psychoticism, PNI Grandiosity and Vulnerability), three out of eight for Mild vs. Moderate PD (PID-5 Negative Affectivity and Detachment, PNI Vulnerability), and one out of eight for Moderate vs. Severe PD (BSL-23 Borderline symptoms; see Table 4).

**Table 4:** Comparisons among the ICD-11 personality disorders degrees of severity on external variables for sample 2 (specialized clinic for less severe personality disorders).

**Scale**	**Statistic**	**Personality difficulty**	**Mild PD**	**Moderate PD**	**Severe PD**	***H***
		**(*n* = 35)**	**(*n* = 95)**	**(*n* = 62)**	**(*n* = 25)**	
SIFS total	*M(SD)*	1.17 (0.08)	1.61 (0.16)	2.15 (0.15)	2.80 (0.22)	191.76^**^
	MR^a^	18.00_a_	83.00_b_	161.59_c_	205.00_d_	
SIFS identity	*M(SD)*	1.82 (0.49)	2.40 (0.53)	2.84 (0.49)	3.23 (0.43)	89.47^**^
	MR^a^	42.90_a_	94.62_b_	141.02_c_	176.76_c_	
SIFS self-direction	*M(SD)*	1.34 (0.52)	1.78 (0.62)	2.24 (0.64)	2.94 (0.64)	75.00^**^
	MR^a^	55.83_a_	93.87_b_	132.06_c_	183.88_d_	
SIFS empathy	*M(SD)*	0.67 (0.34)	1.13 (0.49)	1.58 (0.56)	2.45 (0.63)	98.47^**^
	MR^a^	42.97_a_	93.81_b_	137.03_c_	189.66_d_	
SIFS intimacy	*M(SD)*	0.82 (0.43)	1.10 (0.58)	1.89 (0.50)	2.54 (0.64)	109.17^**^
	MR^a^	55.17_a_	81.52_a_	150.26_b_	186.60_b_	
PID-5 negative affectivity	*M(SD)*	1.51 (0.59)	1.70 (0.56)	1.95 (0.53)	2.22 (0.46)	30.05^**^
	MR^a^	79.01_a_	96.68_a_	126.29_b_	154.90_b_	
PID-5 detachment	*M(SD)*	0.80 (0.50)	1.05 (0.61)	1.43 (0.51)	1.86 (0.53)	53.22^**^
	MR^a^	67.60_a_	93.12_a_	132.58_b_	168.84_b_	
PID-5 antagonism	*M(SD)*	0.49 (0.45)	0.67 (0.60)	0.90 (0.58)	1.17 (0.54)	26.16^**^
	MR^a^	80.51_ab_	97.20_ab_	124.02_bc_	151.68_c_	
PID-5 disinhibition	*M(SD)*	0.83 (0.41)	1.16 (0.48)	1.36 (0.57)	1.67 (0.65)	34.57^**^
	MR^a^	63.34_a_	104.01_b_	126.05_bc_	149.62_c_	
PID-5 psychoticism	*M(SD)*	0.73 (0.44)	1.13 (0.57)	1.22 (0.55)	1.61 (0.73)	27.60^**^
	MR^a^	66.09_a_	108.15_b_	119.07_bc_	147.54_c_	
BSL-23	*M(SD)*	1.39 (0.78)	1.80 (0.84)	1.94 (0.89)	2.69 (0.76)	32.32^**^
	MR^a^	73.84_a_	102.84_ab_	115.82_b_	164.72_c_	
PNI grandiose	*M(SD)*	1.71 (0.86)	2.28 (0.80)	2.44 (0.76)	2.42 (0.81)	16.75^*^
	MR^a^	70.50_a_	111.98_b_	120.28_b_	123.60_b_	
PNI vulnerable	*M(SD)*	1.63 (0.79)	2.24 (0.67)	2.78 (0.72)	3.11 (0.84)	59.15^**^
	MR^a^	55.16_a_	96.48_b_	138.25_c_	159.42_c_	

*ICD-11, 11th revision of the International Classification of Diseases; MR, Mean rank; SIFS, Self and Interpersonal Functioning Scale; BSL-23, 23-item Borderline Symptoms List; PNI, Brief Version of the Pathological Narcissism Inventory; PID-5, Personality Inventory for DSM−5 Brief Form (25 items)*.

a *Mean rank with different subscripts (_a, b, c, d_) indicate significant post-hoc comparisons following a significant Kruskall-Wallis test, two-tailed, p < 0.05, using Bonferroni's correction for multiple comparisons*.

* *p < 0.01*;

** *p < 0.001*.

Comparisons for Samples 3 to 5 did not include the Severe PD category. Again, the expected pattern of increased severity was observed across degrees of severity. In the private practice sample (Sample 3), the following significant contrasts were observed for contiguous categories: two out of eight for No PD vs. Personal difficulty (PID-5 Detachment; PSI symptoms); none between Personality Difficulty and Mild PD, and also none between Mild and Moderate PD. Of note, ECR-12 attachment avoidance did not show any significant difference across degrees of severity (see Table 5). In Sample 4 (pregnant women from the community), significant contrasts for contiguous categories were as follows: six out of seven for No PD vs. Personality Difficulty (K-10 psychological symptoms; EPDS depression; PANAS Negative Affect; DES Absorption and Derealization; PCL-5 trauma); five out of seven between Personality Difficulty and Mild PD (K-10 psychological symptoms; EPDS depression; DES Absorption and Depersonalization; PCL-5 trauma); and none between Mild and Moderate PD (see Table 6). Finally, for Sample 5, significant contrasts for contiguous categories were as follows: six out of 13 for No PD vs. Personality Difficulty (PID-5 Negative Affectivity; IPO Global Personality Organization, Identity Diffusion, and Primitive Defenses; SWLS Satisfaction with life; and RSES Self-esteem); one out of 13 between Personality Difficulties and Mild PD (SWLS Satisfaction with life); and three out of 13 between Mild and Moderate PD (PID-5 Psychoticism; IPO Reality Testing; RSES Self-esteem). IRI Empathic Concern had no discriminant power to distinguish PD degrees of severity (see Table 7).

**Table 5:** Comparisons among the ICD-11 personality disorders degrees of severity on external variables for sample 3 (private practice clinics).

**Scale**	**Statistic**	**No PD**	**Personality difficulty**	**Mild PD**	**Moderate PD**	***H***
		**(*n* = 124)**	**(*n* = 33)**	**(*n* = 64)**	**(*n* = 15)**	
SIFS total	*M(SD)*	0.70 (0.20)	1.17 (0.08)	1.56 (0.17)	2.13 (0.13)	195.70^*^
	MR^a^	62.50_a_	141.00_b_	185.50_c_	229.00_c_	
SIFS identity	*M(SD)*	1.11 (0.51)	1.79 (0.54)	2.17 (0.66)	2.80 (0.48)	116.57^*^
	MR^a^	75.31_a_	140.58_b_	168.77_bc_	212.47_c_	
SIFS self-direction	*M(SD)*	0.83 (0.45)	1.41 (0.39)	1.71 (0.72)	2.25 (0.77)	93.30^*^
	MR^a^	79.20_a_	145.91_b_	161.99_b_	197.59_b_	
SIFS empathy	*M(SD)*	0.43 (0.30)	0.78 (0.46)	1.08 (0.54)	1.70 (0.52)	98.95^*^
	MR^a^	79.83_a_	132.44_b_	164.70_bc_	210.37_c_	
SIFS intimacy	*M(SD)*	0.40 (0.29)	0.72 (0.33)	1.25 (0.58)	1.74 (0.70)	113.11^*^
	MR^a^	77.04_a_	127.33_b_	174.68_c_	202.07_c_	
PID-5 negative affectivity	*M(SD)*	0.95 (0.49)	1.19 (0.57)	1.49 (0.56)	1.71 (0.57)	47.07^*^
	MR^a^	90.96_a_	117.96_ab_	151.67_bc_	174.74_c_	
PID-5 detachment	*M(SD)*	0.37 (0.31)	0.67 (0.48)	0.85 (0.42)	1.29 (0.53)	74.68^*^
	MR^a^	83.06_a_	126.81_b_	158.04_bc_	195.11_c_	
PID-5 antagonism	*M(SD)*	0.42 (0.36)	0.34 (0.30)	0.58 (0.48)	0.76 (0.51)	13.21^*^
	MR^a^	110.48_a_	99.16_a_	131.25_ab_	157.74_b_	
PID-5 disinhibition	*M(SD)*	0.68 (0.44)	0.89 (0.46)	1.14 (0.54)	1.43 (0.71)	39.67^*^
	MR^a^	92.65_a_	120.97_ab_	145.23_b_	169.63_b_	
PID-5 psychoticism	*M(SD)*	0.24 (0.29)	0.34 (0.42)	0.47 (0.39)	0.71 (0.59)	26.23^*^
	MR^a^	98.42_a_	115.23_ab_	144.10_b_	158.79_b_	
PSI total	*M(SD)*	0.86 (0.42)	1.15 (0.46)	1.36 (0.61)	1.64 (0.54)	50.76^*^
	MR^a^	88.72_a_	128.28_b_	147.95_bc_	180.53_c_	
ECR anxiety	*M(SD)*	3.59 (1.23)	4.19 (1.30)	4.84 (1.21)	4.50 (1.72)	37.92^*^
	MR^a^	92.15_a_	120.50_ab_	155.74_bc_	136.53_abc_	
ECR avoidance	*M(SD)^b^*	2.48 (1.08)	2.90 (1.22)	2.81 (1.11)	2.76 (0.96)	4.92

*ICD-11, 11th revision of the International Classification of Diseases; PD, Personality disorder; MR, Mean rank; SIFS, Self and Interpersonal Functioning Scale; PID-5, Personality Inventory for DSM-5 Faceted Brief Form (100 items); PSI, 14-item version of the Psychiatric Symptom Index; ECR, shortened 12-item version of the Experiences in Close Relationships questionnaire*.

a *Mean rank with different subscripts (_a, b, c, d_) indicate significant post-hoc comparisons following a significant Kruskall-Wallis test, two-tailed, p < 0.05, using Bonferroni's correction for multiple comparisons*.

b *Mean rank not shown in the absence of significant contrasts*.

* *p < 0.001*.

**Table 6:** Comparisons among the ICD-11 personality disorders degrees of severity on external variables for sample 4 (pregnant women).

**Scale**	**Statistic**	**No PD**	**Personality difficulty**	**Mild PD**	**Moderate PD**	***H***
		**(*n* = 927)**	**(*n* = 128)**	**(*n* = 126)**	**(*n* = 18)**	
SIFS total	*M(SD)*	0.61 (0.23)	1.14 (0.08)	1.54 (0.16)	2.11 (0.16)	642.23^*^
	MR^b^	464.00_a_	991.50_b_	1118.50_bc_	1190.50_c_	
SIFS identity	*M(SD)*	0.86 (0.38)	1.39 (0.38)	1.97 (0.47)	2.11 (0.51)	436.61^*^
	MR^b^	490.39_a_	865.25_b_	1056.89_bc_	1090.28_c_	
SIFS self-direction	*M(SD)*	0.78 (0.43)	1.37 (0.49)	1.68 (0.51)	2.04 (0.60)	355.76^*^
	MR^b^	500.30_a_	862.90_b_	998.84_bc_	1073.19_c_	
SIFS empathy	*M(SD)*	0.46 (0.33)	0.93 (0.39)	1.28 (0.49)	2.15 (0.57)	392.89^*^
	MR^b^	496.08_a_	867.75_b_	1041.38_c_	1168.14_c_	
SIFS intimacy	*M(SD)*	0.37 (0.31)	0.89 (0.43)	1.25 (0.53)	2.13 (0.45)	394.87^*^
	MR^b^	495.48_a_	876.46_b_	1095.87_c_	1175.67_c_	
K-10	*M(SD)*	2.00 (0.64)	2.33 (0.60)	2.70 (0.63)	2.83 (0.56)	157.00^*^
	MR^b^	519.43_a_	697.26_b_	872.00_c_	945.06_c_	
EPDS	*M(SD)*	1.82 (0.46)	2.13 (0.49)	2.35 (0.52)	2.48 (0.41)	149.60^*^
	MR^b^	524.27_a_	722.01_b_	851.15_c_	942.08_bc_	
PANAS positive affect^a^	*M(SD)*	2.81 (0.62)	2.70 (0.58)	2.61 (0.58)	2.48 (0.41)	18.03^*^
	MR^b^	595.40_a_	528.30_ab_	492.79_b_	380.47_b_	
PANAS negative affect	*M(SD)*	2.17 (0.67)	2.62 (0.72)	2.77 (0.71)	2.87 (0.50)	118.63^*^
	MR^b^	523.55_a_	728.25_b_	802.84_b_	876.15_b_	
DES absorption	*M(SD)*	1.37 (1.19)	2.38 (1.69)	3.07 (1.89)	3.45 (1.88)	155.98^*^
	MR^b^	525.13_a_	716.34_b_	862.23_c_	937.00_bc_	
DES depersonalization	*M(SD)*	0.41 (0.92)	1.10 (1.82)	1.66 (2.18)	2.05 (2.45)	110.66^*^
	MR^b^	542.43_a_	623.52_b_	813.52_c_	857.81_bc_	
PCL-5	*M(SD)*	0.56 (0.51)	0.95 (0.68)	1.27 (0.68)	1.34 (0.57)	174.68^*^
	MR^b^	500.72_a_	723.38_b_	860.57_c_	910.94_bc_	

*ICD-11, 11th revision of the International Classification of Diseases; SIFS, Self and Interpersonal Functioning Scale; MR, Mean rank; K-10, Kessler Psychological Distress Scale; EPDS, Edinburgh Perinatal/Postnatal Depression Scale; PANAS, Positive and Negative Affect Schedule; DES, Dissociative Experiences Scale; PCL-5, Posttraumatic Stress Disorder Checklist for DSM-5*.

a *Higher scores denote better functioning. For all other variables, higher scores denote more severe pathology*.

b *Mean rank with different subscripts (_a, b, c, d_) indicate significant post-hoc comparisons following a significant Kruskall-Wallis test, two-tailed, p < 0.05, using Bonferroni's correction for multiple comparisons*.

* *p < 0.001*.

**Table 7:** Comparisons among the ICD-11 personality disorders degrees of severity on external variables for sample 5 (community participants).

**Scale**	**Statistic**	**No PD**	**Personality difficulty**	**Mild PD**	**Moderate PD**	***H***
		**(*n* = 150)**	**(*n* = 53)**	**(*n* = 45)**	**(*n* = 13)**	
SIFS total	*M(SD)*	0.64 (0.22)	1.15 (0.08)	1.52 (0.14)	2.11 (0.16)	207.28^**^
	MR^b^	75.50_a_	177.50_b_	225.00_c_	255.00_c_	
SIFS identity	*M(SD)*	0.90 (0.41)	1.47 (0.53)	1.85 (0.61)	2.69 (0.44)	118.23^**^
	MR^b^	90.04_a_	95.46_b_	162.74_bc_	246.46_c_	
SIFS self-direction	*M(SD)*	0.83 (0.44)	1.28 (0.40)	1.60 (0.56)	1.94 (0.62)	91.94^**^
	MR^b^	94.38_a_	161.36_b_	191.82_b_	219.23_b_	
SIFS empathy	*M(SD)*	0.43 (0.31)	0.83 (0.41)	1.18 (0.48)	1.48 (0.52)	115.34^**^
	MR^b^	90.71_a_	159.19_b_	205.44_c_	230.15_c_	
SIFS intimacy	*M(SD)*	0.45 (0.39)	1.01 (0.48)	1.43 (0.56)	2.21 (0.45)	129.33^**^
	MR^b^	88.02_a_	164.92_a_	204.11_b_	245.92_b_	
PID-5 negative affectivity	*M(SD)*	0.88 (0.47)	1.24 (0.59)	1.49 (0.61)	1.72 (0.52)	71.71^**^
	MR^b^	87.25_a_	136.72_b_	165.72_bc_	194.86_c_	
PID-5 detachment	*M(SD)*	0.36 (0.34)	0.68 (0.43)	1.02 (0.47)	1.59 (0.18)	95.51^**^
	MR^b^	82.51_a_	126.81_b_	165.85_b_	217.86_c_	
PID-5 antagonism	*M(SD)*	0.46 (0.47)	0.53 (0.39)	0.57 (0.49)	0.70 (0.50)	12.77^**^
	MR^b^	104.76_a_	126.80_ab_	140.26_b_	144.79_ab_	
PID-5 disinhibition	*M(SD)*	0.63 (0.43)	0.90 (0.52)	1.09 (0.53)	1.20 (0.48)	13.33^**^
	MR^b^	104.24_a_	128.66_ab_	139.95_b_	144.46_ab_	
PID-5 psychoticism	*M(SD)*	0.20 (0.29)	0.31 (0.33)	0.46 (0.51)	0.92 (0.60)	35.76^**^
	MR^b^	98.73_a_	129.07_ab_	139.13_b_	196.68_c_	
IRI perspective taking^a^	*M(SD)*	5.30 (0.76)	4.99 (0.87)	4.95 (0.86)	4.34 (1.12)	15.91*
	MR^b^	138.60_a_	115.36_ab_	109.30_ab_	71.89_b_	
IRI empathic concern^a^	*M(SD)^c^*	5.56 (1.12)	5.35 (0.94)	5.54 (0.92)	5.27 (1.07)	3.71
IPO total	*M(SD)*	1.54 (0.27)	1.77 (0.33)	2.02 (0.45)	2.32 (0.55)	74.05^**^
	MR^b^	93.46_a_	142.91_b_	185.12_bc_	201.96_c_	
IPO identity	*M(SD)*	2.07 (0.48)	2.45 (0.54)	2.74 (0.55)	2.89 (0.81)	59.37^**^
	MR^b^	96.54_a_	143.55_b_	175.92_b_	185.32_b_	
IPO defense mechanisms	*M(SD)*	1.46 (0.40)	1.85 (0.58)	2.10 (0.63)	2.59 (0.89)	66.50^**^
	MR^b^	95.69_a_	145.19_b_	173.32_b_	201.64_b_	
IPO reality testing	*M(SD)*	1.19 (0.30)	1.22 (0.21)	1.43 (0.58)	1.71 (0.50)	28.59^**^
	MR^b^	110.59_a_	131.88_ab_	147.50_b_	204.64_c_	
SWLS^a^	*M(SD)*	5.76 (0.90)	5.11 (0.94)	4.25 (1.11)	3.31 (1.42)	86.14^**^
	MR^b^	161.38_a_	113.34_b_	67.90_c_	42.00_c_	
RSES^a^	*M(SD)*	3.49 (0.39)	3.09 (0.40)	2.57 (0.54)	2.33 (0.47)	100.44^**^
	MR^b^	167.50_a_	105.71_ab_	70.58_b_	30.57_c_	

*ICD-11, 11th revision of the International Classification of Diseases; SIFS, Self and Interpersonal Functioning Scale; MR, Mean rank; PID-5, Personality Inventory for DSM−5 Faceted Brief Form (100 items); IPO, Brief 19-item version of the Inventory of Personality Organization; SWLS, Satisfaction with Life Scale; RSES, Rosenberg Self-Esteem Scale*.

a *Higher scores denote better functioning. For all other variables, higher scores denote more severe pathology*.

b *Mean rank with different subscripts (_a, b, c, d_) indicate significant post-hoc comparisons following a significant Kruskall-Wallis test, two-tailed, p < 0.05, using Bonferroni's correction for multiple comparisons*.

c *Mean rank not shown in the absence of significant contrasts*.

* *p < 0.01*;

** *p < 0.001*.

## Discussion

The aim of the present study was to propose an empirically-based classification of the five ICD-11 PD degrees of severity based on the SIFS, a self-report questionnaire that was originally developed to operationalize the level of functioning criterion from the DSM-5 AMPD. Across five clinical and non-clinical samples, we found consistent support for a graduated classification of severity, based on the five categories from the ICD-11 PD model, which range from “No PD” to “Severe PD.” Results tend to support the validity of the proposed anchor points, which were based on the SIFS' total score. They also substantiate the suggestion that diagnostic information from instruments originally developed for Criterion A of the DSM-5 AMPD model can be used with confidence for the ICD-11 PD model's severity classification (13). More generally, our findings also constitute further evidence that general PD severity is a strong predictor of current symptomatology and distress [e.g., (19, 20)].

Prevalence of PD (i.e., as indicated by a score falling within the Mild, Moderate, or Severe PD categories) was very high in PD samples (84.3 and 73.0%), as expected. The prevalence in private practice was 35.1%, with very few participants in the Moderate or Severe categories. This is consistent with the fact that getting treatment in private practice, because of the costs incurred, most generally requires at least some capacity to maintain a source of revenue and thus to be able to sustain employment, which is a characteristic of Mild PD in contrast with the two most severe categories in the ICD-11 model (14). Prevalence in community participants was 12.1% in the sample of pregnant women, and 22.8% in the community sample collected for initial validation of the SIFS; in both cases, severe cases were extremely rare (<1%). The prevalence for the latter sample, while falling within the range (4.4–33.1%) of previous epidemiological studies on PD prevalence, is still noticeably higher than the median of 11.5% reported in a recent review of 14 large-scale studies (67). This result might be partially explained by a self-selection bias, as participants from Sample 5 were aware that they were contributing to a study aiming to validate a self-report pertaining to personality functioning; this may have inadvertently attracted participants with personality issues. Nonetheless, these figures are both in a plausible range, which supports the validity of the proposed cutoff between PD and absence of PD. In addition, results from the ROC curve analysis strengthen findings regarding the validity of the SIFS, which showed an excellent capacity to classify participants from PD groups vs. community samples.

External validation of the five categories from the ICD-11 PD model showed a clear gradient of severity, in all samples, and for virtually all the tested variables; a high number of meaningful differences, both from a statistical and a clinical standpoint, were found. The most striking differences were found in the most severe sample (Sample 1), as there was a very clear delineation among the Mild, Moderate, and Severe categories. These differences were very apparent on external variables reflective of externalizing pathology, notably aggression and impulsivity. This is especially important to support the validity of our classification, as the ICD-11 PD model stresses that the risk of harm to self and others is an important distinction among degrees of severity. It also supports the hypothesis from members of the ICD-11 PD Working Group, which was initially formulated about the borderline PD diagnosis specifically, “that individuals with mild personality disorder will display largely negative affective symptoms, whereas *those with more severe disturbance will also have disinhibited and dissocial behaviors* [emphasis added]” [(4), p. 495]. It also mitigates potential concerns regarding the SIFS' capacity for discrimination based on those variables, as the instrument was primarily developed to assess self and interpersonal dysfunction based on the AMPD model. In non-clinical samples, the most apparent distinctions were observed in Sample 4 (pregnant women), with the cleanest break being observed between the Personality Difficulty and Mild PD degrees; as measures in that sample mostly focused on internalizing pathology (e.g., depression, trauma, negative affectivity, dissociation), these results highlight the SIFS' capacity to discriminate among degrees of severity based on personal distress, which is key to discriminate between Personality Difficulty (where distress is expected to be transient or of low intensity) and Mild PD (where distress may be substantial) in the ICD-11 PD model.

However, it should be noted that the SIFS' capacity for fine-grained discrimination among severity degrees, as measured by the presence of statistically significant differences between contiguous categories, was uneven across samples. Interestingly, the scale's discriminant capacity was at its best in the most severe sample of PD patients (see Table 3), which supports Waugh et al.'s (25) conclusion that the SIFS seems more pathology-focused, and showed a lower discriminant capacity in the private practice sample (see Table 5). One possible—and intriguing—possibility is that “isomorphism” between structural personality deficits assessed by degree of severity measures and external correlates reflective of externalizing and/or internalizing symptoms may be at its highest in clinical samples where patients are the most dysfunctional. In contrast, in private practice patients, there might be a more pronounced gap, as “structural” deficits may be present but with less apparent impact, which is notably reflected in a preserved ability to maintain employment, a prerequisite in most cases to afford psychotherapy in private settings. Other factors, such as issues pertaining to statistical power (with notable differences between group sizes to test for contrasts), also likely played a role.

The main limitation of the present study is the sole reliance on self-reported variables as external validators. While evidence supporting the validity and usefulness of self-ratings of personality pathology is mounting [e.g., (74, 75)], the impact of response style bias as a potential confounding variable could not be taken into account. Future investigations of the validity of the present degrees of severity should not only include multiple instruments, but also multiple methods, as well as longitudinal and behavioral outcomes assessment, most notably to assess the risk of harm to self and others. The lack of uniformity among batteries of measures in the different samples is also a noteworthy limitation, and hampers some potentially useful comparisons. Precise estimation of participants' ethnic background was unavailable, but data were collected in overwhelmingly Caucasian-white communities, which limits generalization of the findings, given than ethnicity has a well-documented impact on PD epidemiology [e.g., (76)]; this calls for inclusion of more diverse samples in future research. There was a significantly unbalanced male-female ratio in the total sample as well as across the four mixed-gender samples (1, 2, 3, and 5).

In sum, the present study provides potentially useful guidelines to determine severity of personality pathology based on the ICD-11 model, using a short self-report questionnaire, the Self and Interpersonal Functioning Scale. Results suggest that the SIFS total score can be used with confidence as a screening tool for provisional assessment of PD severity; it showed a strong capacity, in five independent samples, to classify patients in categories that were meaningfully associated with a number of indices of externalizing and internalizing symptomatology relevant to PD. The present study should be seen as a positive step in the validation of time-efficient, accessible, and cost-effective clinical strategies to overcome the reluctance of some clinicians to diagnose PD, as it is often perceived as difficult, time-consuming, and off-putting [e.g., (29, 77)]. Indeed, the empirically-based anchors presented in this study are straightforward in their interpretation, which is likely to make them appealing for screening purposes to many clinicians irrespective of their PD assessment expertise. In addition, the SIFS provides an opportunity to assess personality pathology in a way that bridges the North American and European emerging conceptualizations of PD severity, with self and interpersonal deficits at their core. In order to rule on the validity of the proposed anchor points, future studies should focus on their usefulness to guide intensity of clinical treatment and to predict treatment course (13).

## Data Availability Statement

The datasets presented in this article are not readily available because Ethics approval did not include sharing of the dataset for the present research. Upon reasonable request, the dataset could be shared, contingent upon the approval of an amendment by ethics committees that authorized the present research. Requests to access the datasets should be directed to dominick.gamache@uqtr.ca.

## Ethics Statement

The studies involving human participants were reviewed and approved by Ethics committees from the Université du Québec at Trois-Rivières, the Université Laval, the Integrated University Health and Social Services Center of the Capitale-Nationale Sectoral Research Ethics Committee in Neurosciences and Mental Health, and the Integrated University Health and Social Services Center of the Mauricie-et-du-Centre-du-Québec. The patients/participants provided their written informed consent to participate in this study.

## Author Contributions

DG and CS designed the study, drafted the first version of the manuscript, analyzed, and interpreted the data. CS drafted the grant proposals that secured financing for the study with the contribution of DG, NB, and MT. CS, NB, and RL drafted the ethics approval demands with the contribution of DG. CS, PL, MP, ML, KM, M-CN, and MT made substantial contributions to data acquisition. PL, NB, AC, JF, RL, KM, and M-CN made substantial revisions to the original draft. All authors have read the manuscript and have approved its submission in the present form.

## Conflict of Interest

The authors declare that the research was conducted in the absence of any commercial or financial relationships that could be construed as a potential conflict of interest.

## Footnotes

^1^In practice, these two groups cannot be perfectly dichotomous and a small overlap between them is expected. On the one hand, a small part of the community sample probably has a diagnosable PD [in line with previous results from large-scale epidemiological studies; (67)]. On the other hand, this is most likely offset by the fact that patients referred for PD treatment might not always have PD proper; they might only display personality difficulty, and instances of misdiagnosis, although rare, cannot be excluded (e.g., Disorders due to substance use or Bipolar type II disorder mimicking PD symptomatology).

^2^Absence of personality difficulty, at the very least, is implausible in these samples. Indeed, all patients from Samples 1 and 2 were referred to their respective clinics after a categorical PD diagnosis, or at least presence of pathological personality traits, was established by a psychiatrist or a general practitioner.
