# Supplementary material for: A Proposed Classification of ICD-11 Severity Degrees of Personality Pathology Using the Self and Interpersonal Functioning Scale
Source: Front Psychiatry. 2021 Mar 18;12:628057. doi: 10.3389/fpsyt.2021.628057 (PMC8012561; doi:10.3389/fpsyt.2021.628057)
Supplement: Supplementary file 1 [file Table_1.pdf]

Table S1

Socio-Demographic Characteristics for the Five Combined Samples

Socio-Demographic Characteristic		Sample 1 (*n* = 287)	Sample 2 (*n* = 249)	Sample 3 (*n* = 242)	Sample 4 (*n* = 1200)	Sample 5 (n = 263)
Age	*M*	33.73	36.87	33.46	29.56	30.57
	SD	10.54	13.49	9.14	4.01	11.86
% Gender	Female	62.0	68.7	63.6	100	73.0
	Male	38.0	30.9	36.4	---	27.0
	Other	---	0.4	---	---	---
% in a relationship		36.2	53.0[a]	70.1	100	Not available
% active (work, school)		47.7	49.0[a]	90.2	79.7[b]	92.1
% Post high-school degree		63.1	79.0[a]	93.0	94.4	95.7

Note. Sample 1 = Specialized psychiatric outpatient clinic for more severe PD; Sample 2 = Outpatient treatment establishments for less severe PD; Sample 3 = Private practice clinics; Sample 4 = Pregnant women; Sample 5 = Community participants; PD = Personality disorder.

[a] Data unavailable for 44 participants.

[b] For Sample 4 (pregnant women), includes preventive withdrawal from work due to pregnancy.
