# Supplementary material for: A Proposed Classification of ICD-11 Severity Degrees of Personality Pathology Using the Self and Interpersonal Functioning Scale
Source: Front Psychiatry. 2021 Mar 18;12:628057. doi: 10.3389/fpsyt.2021.628057 (PMC8012561; doi:10.3389/fpsyt.2021.628057)
Supplement: Supplementary file 2 [file Table_2.pdf]

Table S2

Descriptive Statistics on Personality and Symptoms Measures for the Five Samples

Scale	Statistic	Sample 1 (*n* = 287)	Sample 2 (*n* = 249)	Sample 3 (*n* = 242)	Sample 4 (*n* = 1200)	Sample 5 (*n* = 263)
SIFS Total	*M (SD)*	1.90 (.61)	1.69 (.60)	1.13 (.55)	.79 (.42)	.98 (.49)
	α	.85	.86	.87	.86	.87
SIFS Identity	*M (SD)*	2.55 (.70)	2.37 (.76)	1.63 (.80)	1.05 (.55)	1.29 (.71)
	α	.65	.70	.80	.73	.80
SIFS Self-Direction	*M (SD)*	1.99 (.84)	1.82 (.82)	1.28 (.76)	.96 (.56)	1.11 (.58)
	α	.67	.65	.69	.61	.59
SIFS Empathy	*M (SD)*	1.35 (.78)	1.22 (.74)	.78 (.61)	.62 (.50)	.70 (.52)
	α	.68	.65	.68	.65	.60
SIFS Intimacy	*M (SD)*	1.66 (.89)	1.32 (.81)	.81 (.67)	.55 (.51)	.84 (.69)
	α	.72	.71	.72	.69	.78
PID-5 Negative Affectivity[a]	*M (SD)*	1.88 (.61)	1.73 (.60)	1.19 (.59)		1.39 (.70)
	α	.85	.61	.87		.89
PID-5 Detachment[a]	*M (SD)*	1.45 (.63)	1.15 (.65)	.63 (.50)		.89 (.67)
	α	.85	.64	.86		.90
PID-5 Antagonism[a]	*M (SD)*	.66 (.61)	.71 (.60)	.49 (.43)		.55 (.51)
	α	.90	.72	.86		.88

Scale	Statistic	Sample 1 (*n* = 287)	Sample 2 (*n* = 249)	Sample 3 (*n* = 242)	Sample 4 (*n* = 1200)	Sample 5 (*n* = 263)
PID-5 Disinhibition[a]	*M (SD)*	1.49 (.61)	1.15 (.59)	.92 (.57)		1.03 (.63)
	α	.85	.60	.88		.89
PID-5 Psychoticism[a]	*M (SD)*	.86 (.60)	1.08 (.63)	.37 (.40)		.48 (.52)
	α	.86	.69	.83		.88
BSL-23	*M (SD)*	2.00 (.92)	1.75 (.93)			
	α	.93	.95			
PNI Grandiosity	*M (SD)*	2.30 (.97)	2.17 (.85)			
	α	.85	.78			
PNI Vulnerable	*M (SD)*	2.41 (1.00)	2.25 (.92)			
	α	.88	.86			
BPAQ Trait aggression	*M (SD)*	3.25 (1.12)				
	α	.87				
BPAQ Verbal Aggression	*M (SD)*	2.93 (1.20)				
	α	.60				
BPAQ Physical Aggression	*M (SD)*	2.54 (1.61)				
	α	.86				
BPAQ Hostility	*M (SD)*	3.82 (1.40)				
	α	.69				
BPAQ Anger	*M (SD)*	3.73 (1.46)				
	α	.82				

Scale	Statistic	Sample 1 (*n* = 287)	Sample 2 (*n* = 249)	Sample 3 (*n* = 242)	Sample 4 (*n* = 1200)	Sample 5 (*n* = 263)
IRI Perspective Taking	*M (SD)*	4.54 (1.34)				4.92 (1.05)
	α	.85				.82
IRI Empathic Concern	*M (SD)*	5.39 (1.11)				5.45 (.95)
	α	.78				.77
BIS-11 Attentional	*M (SD)*	2.56 (.51)				
	α	.67				
BIS-11 Motor	*M (SD)*	2.30 (.55)				
	α	.76				
BIS-11 Nonplanning	*M (SD)*	2.55 (.44)				
	α	.70				
PSI Total	*M (SD)*			1.11 (.56)		
	α			.88		
ECR Anxiety	*M (SD)*			4.07 (1.40)		
	α			.86		
ECR Avoidance	*M (SD)*			2.69 (1.12)		
	α			.85		
K-10	*M (SD)*				2.12 (.68)	
	α				.81	
EPDS	*M (SD)*				1.92 (.51)	

Scale	Statistic	Sample 1 (*n* = 287)	Sample 2 (*n* = 249)	Sample 3 (*n* = 242)	Sample 4 (*n* = 1200)	Sample 5 (*n* = 263)
	α				.85	
PANAS Positive Affect	*M (SD)*				2.77 (.62)	
	α				.82	
PANAS Negative Affect	*M (SD)*				2.30 (.71)	
	α				.81	
DES Absorption	*M (SD)*				1.68 (1.48)	
	α				.83	
DES Depersonalization	*M (SD)*				.64 (1.34)	
	α				.76	
PCL-5	*M (SD)*				.70 (.60)	
	α				.92	
IPO Total	*M (SD)*					1.73 (.42)
	α					.82
IPO Identity Diffusion	*M (SD)*					2.34 (.62)
	α					.66
IPO Primitive Defenses	*M (SD)*					1.74 (.62)
	α					.75
IPO Reality Testing	*M (SD)*					1.27 (.40)
	α					.77

Scale	Statistic	Sample 1 (*n* = 287)	Sample 2 (*n* = 249)	Sample 3 (*n* = 242)	Sample 4 (*n* = 1200)	Sample 5 (*n* = 263)
SWLS	*M (SD)*					5.16 (1.26)
	α					.89
RSES	*M (SD)*					3.19 (.57)
	α					.90

Note. Sample 1 = Specialized psychiatric outpatient clinic for more severe PD; Sample 2 = Outpatient treatment establishments for less severe PD; Sample 3 = Private practice clinics; Sample 4 = Pregnant women; Sample 5 = Community participants. SIFS = Self and Interpersonal Functioning Scale; BSL-23 = 23-item Borderline Symptoms List; PNI = Brief Version of the Pathological Narcissism Inventory; PID-5 = Personality Inventory for DSM–5; BPAQ = 12-item version of the Buss-Perry Aggression Questionnaire; IRI = Interpersonal Reactivity Index; BIS-11 = Barratt Impulsiveness Scale (version 11); PSI = 14-item version of the Psychiatric Symptom Index; ECR = 12-item version of the Experiences in Close Relationships questionnaire; K-10 = Kessler Psychological Distress Scale; EPDS = Edinburgh Perinatal/Postnatal Depression Scale; PANAS = Positive and Negative Affect Schedule;. DES = Dissociative Experiences Scale; PCL-5 = Posttraumatic Stress Disorder Checklist for DSM-5; IPO = Brief 19-item version of the Inventory of Personality Organization; SWLS = Satisfaction with Life Scale; RSES = Rosenberg Self-Esteem Scale.

[a] For Samples 1, 3 and 5, the 100-item version of the PID-5 (PID-5 Faceted Brief Form) was used. For Sample 2, the 25-item version (PID-5 Brief Form) was used.
